# Hypoxia and extracellular vesicles: A review on methods, vesicular cargo and functions

**DOI:** 10.1002/jev2.12002

**Published:** 2020-11-14

**Authors:** Nea Bister, Cristiana Pistono, Benjamin Huremagic, Jukka Jolkkonen, Rosalba Giugno, Tarja Malm

**Affiliations:** ^1^ A.I. Virtanen Institute for Molecular Sciences University of Eastern Finland Kuopio Finland; ^2^ Department of Human Genetics KU Leuven Leuven Belgium; ^3^ Department of Computer Science University of Verona Verona Italy; ^4^ Department of Neurology University of Eastern Finland Institute of Clinical Medicine Kuopio Finland

**Keywords:** exosomes, microRNA, hypoxia, cancer, mesenchymal stem cells, infarction, intercellular communication

## Abstract

Hypoxia is an essential hallmark of several serious diseases such as cardiovascular and metabolic disorders and cancer. A decline in the tissue oxygen level induces hypoxic responses in cells which strive to adapt to the changed conditions. A failure to adapt to prolonged or severe hypoxia can trigger cell death. While some cell types, such as neurons, are highly vulnerable to hypoxia, cancer cells take advantage of a hypoxic environment to undergo tumour growth, angiogenesis and metastasis. Hypoxia‐induced processes trigger complex intercellular communication and there are now indications that extracellular vesicles (EVs) play a fundamental role in these processes. Recent developments in EV isolation and characterization methodology have increased the awareness of the importance of EV purity in functional and cargo studies. Cell death, a hallmark of severe hypoxia, is a known source of intracellular contaminants in isolated EVs. In this review, methodological aspects of studies investigating hypoxia‐induced EVs are critically evaluated. Key concerns and gaps in the current knowledge are highlighted and future directions for studies are set. To accelerate and advance research, an in‐depth analysis of the functions and cargo of hypoxic EVs, compared to normoxic EVs, is provided with the focus on the altered microRNA contents of the EVs.

## 1 INTRODUCTION

Correct intercellular communication is needed in diverse cellular processes. Cells can secrete chemical signals which act as local mediators (paracrine model), hormones are released into the circulation and neurotransmitters transmit messages between neurons. Recently, extracellular vesicles (EVs) have emerged as an important additional mechanism of cellular communication and route of interchange of bioactive molecules (Van Niel, D'angelo, & Raposo, 2018).

**FIGURE 1:**
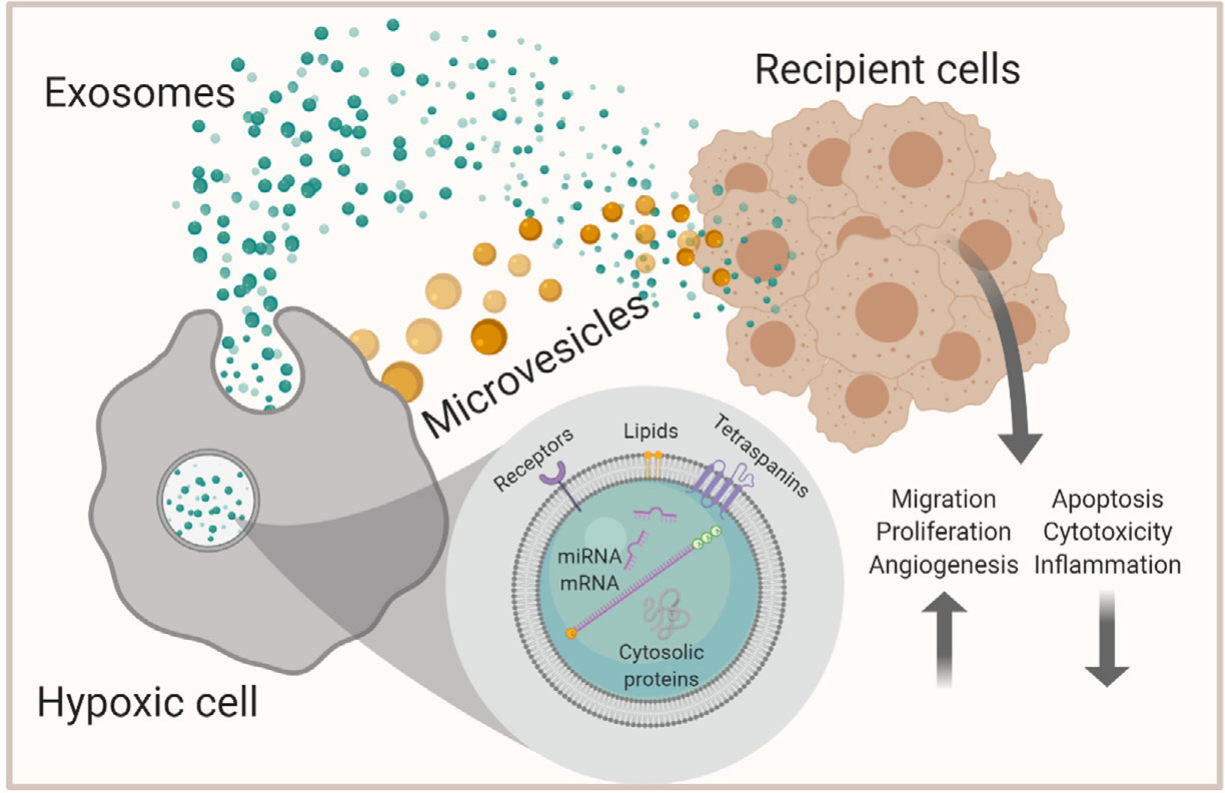
Extracellular vesicle (EV) biogenesis and the properties of hypoxia induced EVs in the recipient cells. Exosomes are formed inside multivesicular bodies (MVBs) by inward budding of the endosomal membrane, leading to the release of small vesicles inside the endosome. MVBs fuse with the plasma membrane to release exosomes to the extracellular space while microvesicles form directly from the outward budding of the plasma membrane. Both EV types contain a variety of cargos. EVs released by hypoxic cells have several functions when they reach the recipient cells. Figure was created with BioRender.com

The term “extracellular vesicle” encompasses a number of biogenetically distinct membranous structures (Kowal et al., 2016; Van Niel et al., 2018). Recently, exosomes, referring to EVs derived from the endocytic cell compartment (Figure 1) after the fusion of the multivesicular bodies with the plasma membrane, have received considerable attention. Exosomes are often referred to as the smallest type of EV, with diameters ranging from 50 to 200 nm. These vesicles are enveloped by a lipid bilayer that consists of molecules present in the plasma membrane of the donor cell, but an enrichment of certain glycoproteins and lipids in the exosome membranes has been observed (Llorente et al., 2013). However, one especially important point worth highlighting in the context of biological processes involving EVs is that exosomes consist of a heterogenous population of vesicles, each likely having a dedicated membrane structure, cargo and function (Van Niel et al., 2018; Willms et al., 2016; Zhang et al., 2018), adding a complete new level of complexity to EV biology. Microvesicles are EVs that, in contrast to the exosomes, directly bud from the plasma membrane of the donor cell and have often been reported to be larger than exosomes (Figure 1), generally between 200 nm and few micrometres, although the size ranges are partly overlapping (Van Niel et al., 2018). Like exosomes, these EVs display a similar level of subtype heterogeneity (Willms et al., 2016). Currently, their biogenesis remains the only apparent difference between exosomes and microvesicles. Thus, after the event of EV release has occurred, these (or other EV subtypes beyond the focus of this review), cannot be distinguished from each other with any level of certainty using current technologies.

One typical obstacle in the field of EV research is the variation in the nomenclature as well as in the study methods being used (Van Deun et al., 2017). Minimal experimental requirements and guidelines for EV research have been published in 2014 (Lötvall et al., 2014) and updated by the International Society for Extracellular Vesicles (ISEV) in 2018 (Théry et al., 2018). In this review, publications are evaluated according to these guidelines. The term *extracellular vesicle* (EV) is used throughout the review to refer to exosomes, microvesicles and other types of EVs (Lötvall et al., 2014).

The hypoxia‐driven effects on EV production and their downstream functions were recently reviewed by Yaghoubi et al. (2020). We will focus our review on technical aspects of EV research and functions of hypoxic EVs (hypo‐EVs) in comparison to normoxic EVs (normo‐EVs). In addition, we have conducted a bioinformatic analysis which revealed novel information on the several biological pathways affected by several hypo‐EV associated miRNAs. Only publications where a distinct hypoxic *in vitro* or *ex vivo* insult had been utilized were included in this review, i.e. we have excluded experiments where EVs from biological fluids were isolated after hypoxic or ischemic injury *in vivo*. These *in vivo* experimental designs measure effects that are a mixture of several types of exposures, such as inflammation together with hypoxia, making it hard to interpret the data only in the context of hypoxia. Indeed, it has been reported that inflammatory and hypoxic insults induce the release of EVs with different and distinct cargos from endothelial cells (De Jong et al., 2012). A total of 70 publications matching our criteria were evaluated in this review. Finally, we have considered future prospects for the field of hypoxic EV research to facilitate follow‐up research.

## 2 HYPOXIA AND EXTRACELLULAR VESICLES

Hypoxia refers to a decline of tissue oxygen saturation. Brain and limb ischemia (Guglielmotto et al., 2009), myocardial infarction (MI) (Greco, Gaetano, & Martelli, 2014), pulmonary disease (Lee, Ko, Ju, & Eltzschig, 2019) and obesity (Goossens & Blaak, 2015) are common pathological conditions related to hypoxia. In cancer tissue, hypoxia is caused by the increased oxygen consumption or outgrowth of the blood supply due to the high proliferation of cells (Jain, 2014; Palazon et al., 2017; Rapisarda & Melillo, 2012).

Hypoxia activates the hypoxia inducible factor (HIF) signalling pathway (LaGory & Giaccia, 2016; Semenza, 2000). Under normoxic conditions, HIF‐α is rapidly hydroxylated by prolyl‐4‐hydroxylases (PHDs) and directed to proteosomal degradation. When there is hypoxia, however, this degradation process is suppressed, and the HIF‐α subunits translocate into the nucleus to bind with HIF‐1β (HIF1B). The heterodimeric complex then locates to the hypoxia‐responsive elements (HREs) leading to the subsequent upregulation of more than 100 target genes including vascular growth factors VEGF‐A and PDGF‐B to promote tissue survival (Pugh & Ratcliffe, 2003). In addition, there are HIF‐independent signalling pathways that are activated under hypoxia such as the nuclear factor‐κB (NF‐κB) (Rius et al., 2008), mTOR (Hudson et al., 2002) and STAT3 (Noman et al., 2009) pathways.

**FIGURE 2:**
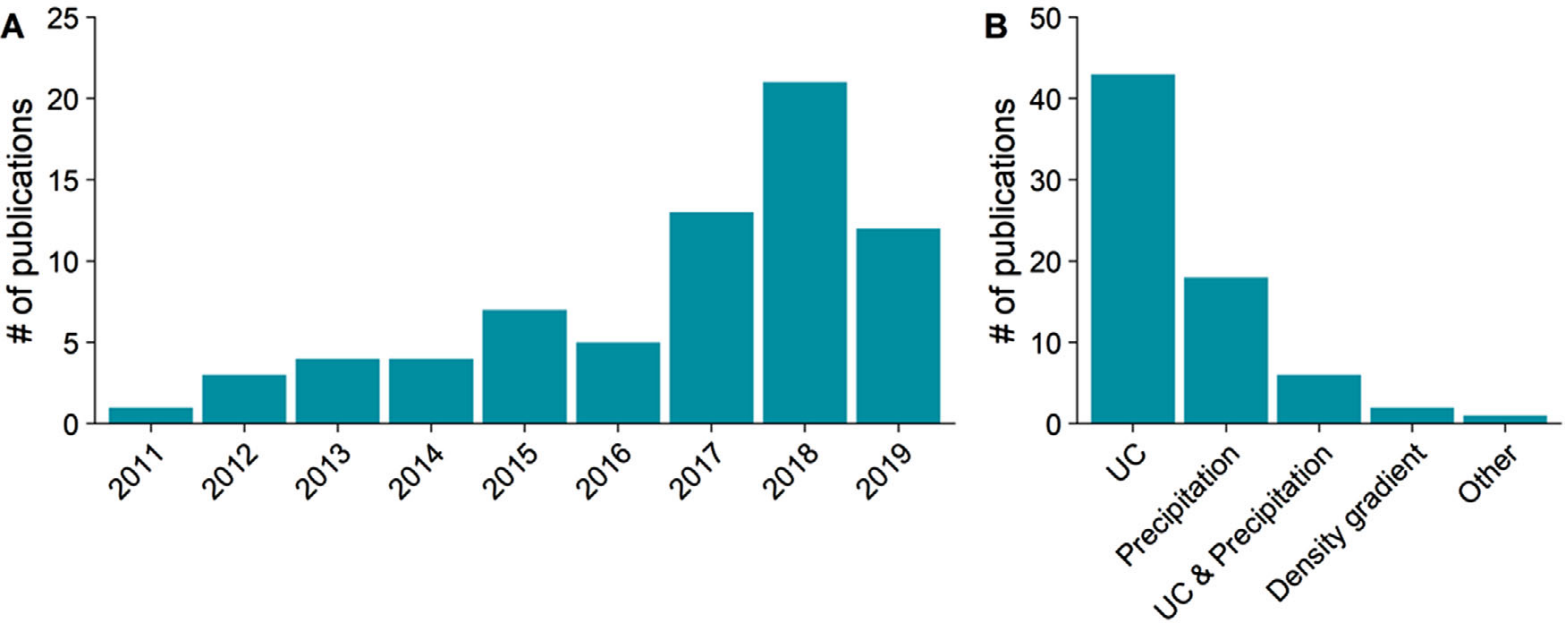
Number of publications on hypoxia induced extracellular vesicles (EVs) is rapidly accumulating and shows significant heterogeneity in the EV isolation methods used. Number of new publications on hypo‐EVs has been increasing during the last few years (a). Numbers are based on PubMed searches using “extracellular vesicle”, “exosome” or “microvesicle” and “hypoxia” or “ischemia” search words and screening for suitable articles based on the title which yielded 70 original publications. In these publications, different techniques were used for EV isolation, most commonly involving ultracentrifugation (UC) or precipitation‐based methodologies (b). Gradient refers to the use of an iodixanol or sucrose (or some other agent) density gradient step in the UC purification

Much effort has been exerted to understand how to control the complex effects of hypoxia, as ways to promote tissue survival and recovery, and to prevent disease propagation. Hypoxia‐induced harmful and beneficial effects are both mixed and difficult to distinguish. For example, hypoxia induces vascularization in cancer tissue and a progression of the disease (Jain, 2014). On the other hand, penumbral angiogenesis is associated with the functional recovery encountered in stroke patients (Krupinski, Kaluza, Kumar, Kumar, & Wang, 1994). Hypoxic preconditioning of mesenchymal stem cells (MSCs) improves their efficacy in the treatment of cardiovascular diseases (Hu et al., 2016; Wang et al., 2017). Interestingly, EVs from the hypoxia preconditioned MSCs alone are sufficient to reduce the infarct sizes after an MI (Feng, Huang, Wani, Yu, & Ashraf, 2014). It has also been suggested that remote ischemic preconditioning induces the release of EVs that are protective in cerebral ischemia (Li et al., 2019) and kidney ischemia/reperfusion injury (Zhang et al., 2019). In *in vivo* conditions, hypoxia affects several types of cells, and cell‐to‐cell communication plays a vital role in the outcome of this interplay. The recent interest in EVs as carriers of functional signalling molecules between different cell types provides a compelling mechanism to account for many of the hypoxia‐induced cellular responses. Indeed, the literature on the release of EVs in response to hypoxia has been rapidly accumulating (Figure 2a).

The biogenesis of EVs has been extensively reviewed elsewhere (Abels & Breakefield, 2016; Van Niel et al., 2018). One of the known mechanisms for EV biogenesis involves the recruitment of the membrane anchored Ras superfamily of small G proteins (RABs) for membrane budding and fusion processes. There are indications that induction of HIFs could directly affect the EV biogenesis pathways involving RABs. Hypoxia was shown to induce the EV release from breast cancer cell lines and this induction was lost when HIF1 or 2α were silenced, suggesting that the release of the EVs was HIF dependent (Wang et al., 2014). In addition, HIFs were shown to directly bind to the *RAB22A* locus and to induce the expression of RAB22A, a protein required for the budding of the microvesicles from the plasma membrane. By silencing RAB22A expression, the hypoxia‐induced EV release was prevented. Direct binding of HIF1α to the *RAB27A* promoter has been described in B cells and the observed increased release of EVs due to hypoxia was shown to be dependent on this process (Zhang et al., 2019). In addition, hypoxia induced the release of EVs from ovarian cancer cells possibly by altering the levels of RABs (Dorayappan et al., 2018). It has been suggested that decreased Rab7 could promote fusion of MVBs with lysosomes, leading to the degradation of vesicles, while increased levels of Rab27a could promote plasma membrane fusion under hypoxic conditions. However, the exact mechanisms controlling the fate of MVBs, or the release of the EVs, in response to hypoxia in other cellular contexts still remain to be clarified. Adding to the directly EV‐linked evidence, other RABs have been shown to respond to hypoxia, i.e. hypoxia induced the expression of RAB11, and the silencing of RAB11 prevented the hypoxia‐induced migration and invasion of HeLa and SiHa cells (Xu et al., 2017). It is known that RAB11 is required also for the upregulation of matrix metalloproteinases in response to hypoxia, meaning that the processes described for the EV biogenesis are widely connected to several important cellular processes involving the movement and the reorganization of cellular membranes.

Another distinct mechanism for exosome biogenesis involves the synthesis of ceramide from sphingomyelin by neutral type II sphingomyelinase (nSMase2). Knocking out of nSMase2 reduces the release of EVs from neuron‐like cells (Sackmann et al., 2019). However, hypoxia reduced the expression of nSMase2 while increasing the release of EVs, suggesting that the hypoxia‐induced EV release occurred via nSMase2‐independent mechanisms. Interestingly, inhibition of nSMase2 exacerbated hypoxia‐induced death of neurons, closely connecting the EV biogenesis processes to the regulation of cell viability. Some evidence points towards the involvement of nSMase2 activation in apoptotic processes (Shamseddine, Airola, & Hannun, 2015), but the upstream regulation of ceramide synthesis in response to hypoxic stimulus was reported to be highly complex and possibly cell type dependent (Kang et al., 2010). However, to understand hypoxia‐induced EV signalling, it is crucial to understand the relationships between hypoxia, apoptosis, EVs and ceramide pathways. Apoptotic bodies, and other vesicles released from the cells undergoing apoptosis, are EVs that have received less attention in the field of EV research (Poon et al., 2019). This group of vesicles is highly heterogeneous and has been suggested to transport functional molecules which can elicit reparative functions in the recipient cells similarly to other EVs (Hristov, Erl, Linder, & Weber, 2004; Zernecke et al., 2009). Future studies should focus on the effects of hypoxia on the formation of apoptotic vesicles and on cell viability from a wider perspective.

## 3 METHODS FOR EV ISOLATION AND CHARACTERIZATION

EVs can be isolated by several different methods such as ultracentrifugation (UC), density gradients, commercial kits, immunoaffinity‐based protocols, and size‐exclusion chromatography (SEC) (Théry et al., 2018). The choice of isolation method has a significant impact on the purity of EV preparations, as well as on which vesicle populations will be recovered (Royo et al., 2016). It may also affect the functionality of EVs (Takov, Yellon, & Davidson, 2019). Recent studies support the use of density gradient‐ and SEC‐based methods that tend to yield EVs of high purity (Théry et al., 2018). However, the obtained yield and purity are directly dependent on the type of sample from which the EVs are being enriched. While Tian et al. (2020) demonstrated higher purity of plasma EVs isolated by UC compared to any commercial kits, including SEC columns, Benedikter et al. (2017) reported that for cell conditioned culture medium, SEC achieves a superior yield and less variation as compared to UC. Furthermore, non‐vesicular extracellular cytokines have been shown to co‐isolate with UC purified EVs from melanoma cell lines (Shu et al., 2020). This cytokine contamination was significantly reduced by SEC, suggesting that SEC could achieve superior purity for cell culture derived EVs. The use of SEC for the EV enrichment is also supported by its convenience as the non‐EV fractions from the same samples can be concomitantly analysed for additional evidence of the association of the EVs with other molecules under study (Benedikter et al., 2017). Some advantages and disadvantages of the common EV isolation methods are presented in Table 1 with a focus on cell culture enriched EVs.

**TABLE 1:** Aspects of the beneficial and limiting characteristics of the most widely applied techniques for the enrichment of extracellular vesicles

Extracellular vesicle enrichment method	Advantages	Disadvantages	References
Ultracentrifugation	Moderate purity and yield of EVs	Time consuming, special equipment required, risk of EV aggregation and operator dependent variation	Benedikter et al. (2017)), Linares, Tan, Gounou, Arraud, and Brisson (2015))
Density gradient	High purity	As for ultracentrifugation + low yield	Konoshenko, Lekchnov, Vlassov, and Laktionov (2018)
Precipitation‐based	Fast, high yield	Very low purity, commercial kits do not report exact chemical components	Tian et al. (2020))
Size‐exclusion chromatography	Fast, moderate/high purity, yield and functionality of EVs	Additional concentration steps prior and after may be required	Benedikter et al. (2017))
Ultrafiltration	Fast, no expensive equipment	Low purity when used alone, EVs can bind to the membrane reducing yield	Benedikter et al. (2017)), Konoshenko et al., 2018)

For complex biofluids, such as plasma, combinations of several isolation methods are being continuously developed to collect EVs of higher purity. Recently, Zhang, Borg, Liaci, Vos, and Stoorvogel (2020) proposed combining PEG polymer precipitation with an iohexol density gradient and then SEC, in that order. A minimal loss of EVs was observed after precipitation, suggesting it as a feasible upstream sample preparation step for inclusion in many EV purification techniques, as an initial concentration step is often required to decrease the sample volume. The addition of steps to fractionate the samples based on density and size by iohexol gradient and SEC, respectively, resulted in the efficient elimination of non‐EV material such as lipoprotein particles and protein aggregates, in comparison to any of the purification steps alone. While a similar approach had been applied before and also highlighting the crucial interference by lipoproteins, it did not include the initial precipitation step and reported the starting plasma volume as 6 ml (Karimi et al., 2018). This can be compared to the 1 ml of plasma utilized for the protocol with the PEG precipitation and the very impressive recovery rate of spike‐in B cell derived EVs reported (Zhang, Borg, Liaci, Vos, & Stoorvogel, 2020).

In addition to the varying yields and contaminant profiles resulting from different EV isolation techniques, different methodological approaches can result in the enrichment of different EV populations. Indeed, the comparison of the recovery level of different tetraspanins, and other proteins commonly known to be enriched in EVs, showed highly variable profiles between different EV enrichment techniques (Tian et al., 2020). These techniques involved different precipitation and membrane affinity based commercial kits, SEC and UC, suggesting that all of the methods have EV subpopulation dependent enrichment efficiencies.

Regarding the literature on hypo‐EV studies, most of the evaluated studies have utilized UC (70%) or commercial precipitation (24%) based methods (Figure 2b), the former being considered as a “golden standard” method for EV isolation (Théry, Amigorena, Raposo, & Clayton, 2006). Even more variation was observed in the extensiveness of the characterization of isolated EVs between different publications, suggesting that the materials being analysed in various publications may in fact differ significantly from each other, an issue that is extensively discussed in the field of EV research.

## 4 METHODS FOR HYPOXIA INDUCTION

**FIGURE 3:**
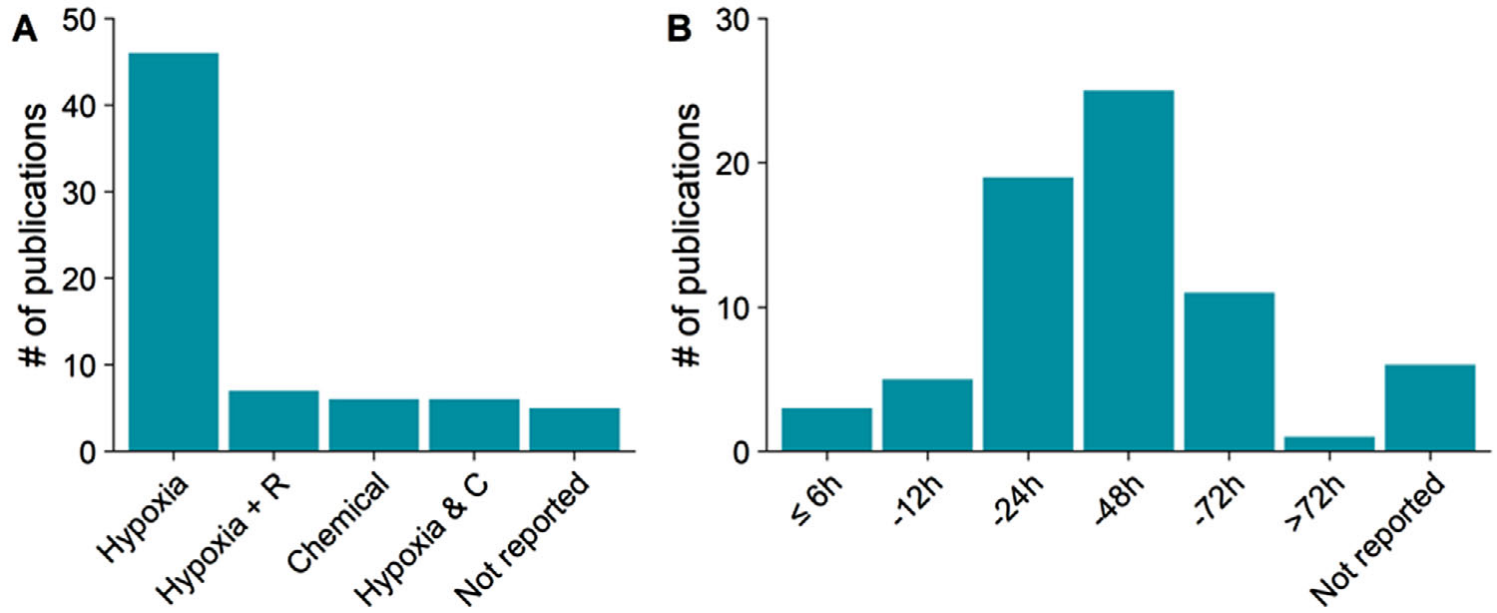
Experimental set‐ups differ by the method of hypoxia induction and the length of this exposure. Most studies were carried out using a hypoxic incubator with low oxygen levels (a). Some studies used both hypoxia and chemical hypoxia. The duration of hypoxia was commonly between 24 and 48 h (b). For reoxygenation studies, the duration of reoxygenation is plotted. R = reoxygenation, C = chemical

There are several ways to mimic a hypoxic insult *in vitro*, introducing another crucial variable to the study design. The most widely used method in the reports reviewed here has been incubation with variable oxygen levels (Figure 3a). In addition, chemical hypoxia has been used in several studies. While being convenient, the chemicals used for hypoxia induction, such as cobalt chloride (De Jong et al., 2012), do not completely mimic the effects and dynamics of the lack of oxygen (Holloway & Gavins, 2016). In addition to the method of hypoxia induction, there is extensive variation in the length of the exposure to hypoxia, ranging from half an hour up to 6 days (Figure 3b). In the field of cardiovascular diseases, it is common that cells are submitted to reoxygenation of variable lengths after hypoxia and the cellular pathways, and thus the outcome of this hypoxia/reoxygenation injury, has been shown to differ extensively from hypoxia without reoxygenation (Kalogeris, Baines, Krenz, & Korthuis, 2012). A remarkable fraction of studies in fact failed to report any details on how hypoxia was induced.

## 5 EFFECTS OF HYPOXIA ON CELL VIABILITY AND EV RELEASE

**FIGURE 4:**
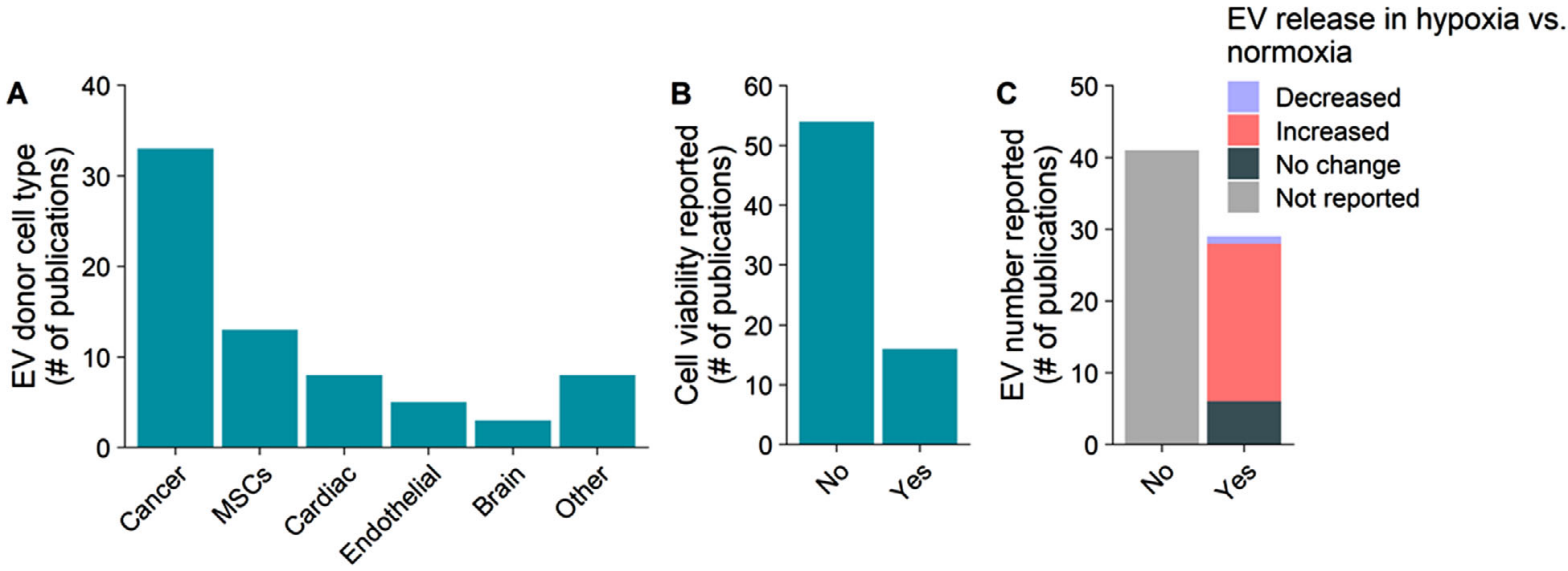
Effect of hypoxia on extracellular vesicle (EV) release. The effect of hypoxia on the release of EVs has been studied in the context of several different EV producing cell types (a). Only 1/5 of publications reported the effect of hypoxia induction on the viability of the EV producing cells (b). Studies reporting quantitative effects on EV release reported mainly increased EV release during hypoxia, or no change (c)

Almost half of the studies analysed hypo‐EVs from different types of cancer cells while hypo‐EVs from MSCs and cardiac cells were the second and third most widely studied cell types (Figure 4a). Different cell types have major differences in their tolerance to the lack of oxygen (Salomon et al., 2013; Yang et al., 2016). Most of the analysed studies did not report any measure of cell viability during EV collection (Figure 4b), and even those that did, showed either unaltered or decreased viability. In the analysis of EVs from cell cultures, it is advised that the viability of cells should be high and the number of dead cells should be reported (Théry et al., 2018). Increased cell death can potentially introduce significant contaminations to the EV preparations and can alter the EV populations being recovered.

Quantitative measurement of EVs has found evidence of increased EV release after hypoxia as compared to normoxia (Figure 4c). EV release was measured by several different methods, such as by total protein, the quantity of EV‐enriched markers or the number of particles, all of which are common approaches used in the literature (Hartjes, Mytnyk, Jenster, Van Steijn, & Van Royen, 2019). None of these methods directly or exclusively measure EVs for example, they can be compromised by the levels of contaminating proteins between hypo‐EVs and normo‐EVs. Indeed, the real meaning of these observations is uncertain and they have been interpreted differently by different authors. However, over half of the studies did not comment on this issue and the differences in their interpretation can be proposed as a reason why some authors refrained from stating any effect of hypoxia on EV release. Despite the above‐mentioned issues, the reports seem to be surprisingly consistent, supporting the hypothesis that EV release is induced upon hypoxia. Nonetheless, the apparent increase in EV release measured by specific protein abundances, such as tetraspanins, could be due to a higher loading of these proteins per vesicle while absolute EV number would not change. While cells secrete heterogeneous populations of EVs, hypoxia could lead to the secretion of vesicles with different physical characteristics than in basal conditions and mechanistic EV biogenesis studies in the context of hypoxia are still lacking.

Only one study reported a decreased release of EVs as measured by Nanoparticle Tracking Analysis (NTA) from endothelial colony forming cells after 72 h conditioning at 1% O_2_ (Liu et al., 2018). In addition, a reduced level of cellular nSMase2, a protein required for the release of at least some EV subtypes (Van Niel et al., 2018), was reported. Other studies evaluating endothelial cell derived EVs have either not quantified EV release (De Jong et al., 2012; Jong, Balkom, Gremmels, & Verhaar, 2016; Zhang et al., 2016) or detected increased release (Burnley‐Hall, Willis, Davis, Rees, & James, 2017). Increased release was observed after considerably shorter hypoxia times, only 24 h. Since so few studies reported both donor cell viability and quantitated EV release, it is not possible to state whether there is a correlation between cell death and the level of EV release.

In summary, most studies report increased release of EVs in response to hypoxia, highlighting the need of stable controls for normalization. Currently, it remains unclear if the effects observed here are caused by an increased number of released EVs in hypoxia, an altered content within the individual EVs, or both.

## 6 EFFECTS OF HYPOXIC EVs ON RECIPIENT CELLS

Functional outcomes of hypo‐EVs were evaluated based on the recipient cell types, i.e. the cell type being exposed to the EVs isolated from hypoxic donor cells. This approach was used because in similar recipient cell types similar outcomes are more frequently being examined and make possible a reasonable comparison between different reports.

### 6.1 Endothelial cells

Hypo‐EVs from distinct cell types such as different cancer cells, MSCs and cardiac cells showed highly consistent functional features when endothelial cells were exposed to these EVs (Table 2). Nine out of 13 studies used HUVEC cells as recipients for hypo‐ and normo‐EVs and they consistently showed increased survival of the hypo‐EV exposed cells as compared to normo‐EVs exposed cells. Most publications report the superior angiogenic capacity of hypo‐EVs over normo‐EVs. Indeed, EV release has been speculated to be one of the mechanisms behind the survival and promotion of cancer in which lack of oxygen is a crucial factor when the size of the tumour grows, requiring the formation of new blood vessels (Muz, De La Puente, Azab, & Azab, 2015). Ontoria‐Oviedo et al. (2018) suggested that the angiogenic capacity of cardiomyocyte hypo‐EVs could be species dependent, causing the observed anti‐angiogenic effect as observed in a human cardiac cell line. They also detected a higher uptake of cardiomyocyte EVs in endothelial cells as compared to fibroblasts and suggested that the differences in the functions of EVs in distinct recipient cell types could be related to the differential EV uptake capacities of the cells.

**TABLE 2:** Functional outcome of exposure of endothelial cells to either hypo‐EVs or normo‐EVs

EV donor	Donor cell species	Donor cell model	Type of hypoxia	Angiogenesis	Apoptosis/cell death	Migration	Proliferation	Reference
Adipose‐derived MSCs	Human	Primary	H	+		+		Han et al. (2018))
Adipose‐derived MSCs	Human	Primary	H	+				Xue et al. (2018))
Adipose‐derived MSCs	Human	Primary	H	+				Collino et al. (2019))
Placental MSCs	Human	Primary	H	+		+	+	Salomon et al. (2013))
Cardiac progenitor cells	Rat	Primary	H	+				Gray et al. (2015))
Cardiomyocytes	Human	Secondary	H	+				Ontoria‐Oviedo et al. (2018))
Cardiosphere‐derived cells	Human	Primary	H	+				Namazi et al. (2018))
Colorectal cancer	Human	Secondary	H			+	+	Huang and Feng (2017)
Glioblastoma	Human	Secondary	H	+			+	Svensson et al. (2011))
Glioblastoma	Human	Secondary	H		‐			Zhang et al. (2017))
Glioblastoma	Human	Secondary	H	+	‐		+	Kucharzewska et al. (2013))
Hepatocellular carcinoma	Human	Secondary	C	+				Sruthi et al. (2018))
Liver cancer	Human	Secondary	H	+				Matsuura et al. (2019))
Lung cancer	Human	Secondary	H	+		+		Hsu et al. (2017))
Lung cancer	Human	Secondary	H	+		+		Hsu et al. (2018))
Leukaemia	Human	Secondary	H	+	No effect		No effect	Tadokoro et al. (2013))
Multiple myeloma	Human	Secondary	H	+				Umezu et al. (2014))
Nasopharyngeal carcinoma	Human	Secondary	H	+		+	+	Shan et al. (2018))
Pericytes	Human	Primary	C	+				Mayo and Bearden (2015)

Hypoxia exposed cells release EVs with pro‐angiogenic capacity. These EVs were shown to promote cell survival, migration, and proliferation of endothelial cells. H, hypoxia, C, chemically induced hypoxia, +/‐, increased/decreased by hypo‐EVs compared to normo‐EVs, grey, not studied; MSC, mesenchymal stem cell.

### 6.2 Cardiomyocytes

We found a total of six papers in which cardiomyocytes had been exposed to hypo‐EVs isolated from either cardiac cell types, endothelial cells or MSCs (Table 3). In these papers, hypoxia/reoxygenation induced EVs consistently evoked propagated cell death and apoptosis in cardiomyocytes independent of the cell type producing the EVs, in agreement with earlier speculations. However, also the study of Yu et al. (2012) detected decreased cell survival of cardiomyocytes after exposure to hypo‐EVs from cardiomyocytes without reoxygenation, suggesting that the effect may be dependent on the EV‐producing cell type. This is indeed supported by two recent observations that MSC derived hypo‐EVs exerted a protective effect against cell death in cardiomyocytes (Table 3).

**TABLE 3:** Functional outcome of cardiomyocytes exposed to either hypo‐EVs or normo‐EVs

EV source	Donor cell species	Donor cell model	Type of hypoxia	Apoptosis/cell death	Reference
Cardiac fibroblasts	Mouse	Primary	H/R or H	+/‐ *	Cosme, Guo, Hadipour‐Lakmehsari, Emili, and Gramolini (2017))
Cardiomyocytes	Rat	Secondary	H/R	+	Wang, Yuan, Yang, and Li (2017)
Cardiomyocytes	Rat	Primary	H or C	+	Yu et al. (2012))
Endothelial cells	Human	Primary	H/R	+	Zhang et al. (2016))
MSCs	Human	Primary	H	‐	Park et al. (2018))
MSCs	Mouse	Primary	H or C	‐	Zhu et al. (2018))

Hypoxia followed by reoxygenation transforms released EVs into pro‐apoptotic when administered on cardiomyocytes. Hypoxia alone produces effects consistent with other cell types, promoting cell survival. H, hypoxia, H/R, hypoxia/reoxygenation, C, chemically induced hypoxia, +/‐, increased/decreased by hypo‐EVs compared to normo‐EVs, *, increased apoptosis with H/R EVs, MSC, mesenchymal stem cell.

Eguchi et al. (2019) tested different mechanisms of EV uptake into cardiomyocytes and showed that hypoxia induced the uptake of EVs, mainly due to increased clathrin‐dependent endocytosis. This hints at a potential approach to manipulate the composition or environment of EVs to guide therapeutic agents via these structures to only those cells needing the treatment. Indeed, methods to conjugate the EV membrane with specific tags to escort EVs to certain target tissues have been already demonstrated in the case of heart and brain (Tian et al., 2018; Zhu et al., 2018). Further research is required to validate whether the cellular uptake of hypo‐EVs is higher than in normo‐EVs, and if so, how does the hypoxic condition modulate the EVs’ composition, leading to their increased uptake and which factors mediate this phenomenon.

To conclude, similar functions can be observed with hypo‐EVs across different donor cell types. However, the functions of hypo‐EVs may not be completely independent of the EV producing cell type as exceptions to this rule were demonstrated, especially in cardiomyocytes. To better understand this phenomenon, additional studies on hypo‐EVs especially from non‐proliferating cell types would be beneficial.

### 6.3 Cancer cells

Cancers are disorders where uncontrolled cell division leads to a malignant growth of the tissue. This division may be caused by mutations in the genes controlling these processes. Normally these mutations occur and altered cells are removed by several mechanisms. However, sometimes these malignant cells avoid the growth control mechanisms and the cancer continues to develop. Thus, cancer cell encompasses a group of highly heterogenous cells and this diversity is observed even inside a single tumour (Fisher, Pusztai, & Swanton, 2013).

Published data consistently show induced migration and an elevated invasion ability of hypo‐EV‐treated cancer cells as compared to normo‐EV‐treated cells, regardless of the cancer type (Table 4). This observation supports the hypothesis that EVs may play a key role in cancer metastasis as has been speculated in several studies (An et al., 2015). Zhao et al. observed increased cancer cell proliferation and blood‐brain barrier permeability after treatment with glioblastoma hypo‐EVs, effects which could support brain cancer progression and metastasis (Zhao, Wang, Xiong, & Liu, 2018). The effect was not reproducible with hypo‐EVs from HEK293 cells, evidence of cell type specificity in the hypoxic response. Ren et al. detected a similar proliferative capacity with hypo‐EVs from bone marrow‐derived MSCs (Ren et al., 2019). This suggests that it is not only malignant cells that can secrete pro‐proliferative EVs under hypoxic conditions, but the effect may be at least partly dependent on the donor cell's proliferative capacity.

**TABLE 4:** Functional outcome of cancer cells exposed to either hypo‐EVs or normo‐EVs

EV source	Donor cell species	Donor cell model	Type of hypoxia	Migration/invasion	Proliferation	Stemness	Reference
Bone marrow derived MSCs	Human	Primary	H	+	+	+	Ren et al. (2019))
Bone marrow derived MSCs	Human, mouse	Primary	H	+		+	Zhang et al. (2019))
Bladder cancer	Human	Secondary	H	+	+		Xue et al. (2017))
Breast cancer	Human	Secondary	H	+			Wang et al. (2014))
Erythroleukaemia	Human	Secondary	H			+	Shi et al. (2017))
Glioblastoma	Human	Secondary	H		+		Zhao et al. (2018))
Nasopharyngeal carcinoma	Human	Secondary	H or C			+	Shan et al. (2018))
Oral squamous cell carcinoma	Human	Secondary	H	+			Li et al. (2016))
Ovarian cancer	Human	Secondary	H	+			Dorayappan et al. (2018))
Prostate cancer	Human	Secondary	H	+			Schlaepfer et al. (2015))
Prostate cancer, prostate stroma	Human	Secondary	H	+		+	Ramteke et al. (2015))

Effects of hypo‐EVs on cancer cells have been evaluated by measuring the migratory, invasion and proliferative capacity of EV exposed cells. The literature is consistent in showing effects suggesting cancer promoting effects. H, hypoxia, C, chemically induced hypoxia, +/‐, increased/decreased by hypo‐EVs compared to normo‐EVs, grey, not studied, MSC, mesenchymal stem cell.

From the uncontrollable proliferation of the cancerous cells, it follows that the cells start to resemble MSCs, which have the capability to continuously divide and differentiate into several different cell types, more than the dedicated, terminally differentiated, cell types. This is called the epithelial–mesenchymal transition (EMT) and it is characterized by certain changes in the gene expression such as increased expression levels of vimentin but decrease in E‐cadherin. All studies evaluating the effect of hypo‐EVs on recipient cell stemness have reported enhanced EMT (Table 4). The evidence strongly supports the observation that EVs resemble their donor cells (Sork et al., 2018), but the current literature on functions of hypo‐EVs in cancer cells is limited to MSCs, cancer cell and embryonic kidney cell‐derived EVs whilst nothing is known about hypo‐EVs from cells that exhibit fewer features of stemness. Furthermore, all immortalized continuous secondary cell lines exhibit cancer‐like properties which limit their use, highlighting the need for studies exploiting more representative models.

### 6.4 Immune cells

There are an increasing number of studies reporting that hypo‐EVs are anti‐inflammatory when immune cells are exposed to these structures as compared to normo‐EVs (Table 5). Interestingly, this effect was opposite in the study of Yang et al. (2018); these investigators described a pro‐inflammatory phenotype of bone marrow‐derived neutrophils after exposure to EVs isolated from a hypoxia/reoxygenation injury. This suggests that the reoxygenation has a significant role in the functions of the released EVs, similarly to distinct responses seen at cellular level. However, to our knowledge, this study remains the only one investigating the abilities of hypoxia/reoxygenation induced EVs to evoke immune cell polarization.

**TABLE 5:** Functional outcome of immune cells exposed to either hypo‐EVs or normo‐EVs

EV source	Donor cell species	Donor cell model	Type of hypoxia	Anti‐inflammatory	Pro‐inflammatory	Cytotoxic activation	Reference
Bone marrow derived MSCs	Mouse	Primary	H	+			Cui et al. (2018))
Bone marrow derived MSCs	Human	Primary	H	+			Li, Jin, and Zhang (2015))
Bone marrow derived MSCs	Human	Primary	H	+			Ren et al. (2019))
Glioblastoma	Human, mouse	Secondary	H	+		‐	Guo et al. (2018))
Hepatocytes	Mouse	Secondary	H/R		+	+	Yang et al. (2018))
Lung cancer	Human	Secondary	H	+			Hsu et al. (2017))
Oral squamous cell carcinoma	Human	Secondary	H			‐	Li et al. (2019))
Ovarian cancer	Human	Secondary	H	+			Chen et al. (2017))
Ovarian cancer	Human	Secondary	H	+			Chen et al. (2018))
Pancreatic cancer	Human	Secondary	H	+			Wang et al. (2018))
Tumour cells	Human	Secondary	A			‐	Berchem et al. (2016))

Hypo‐EVs promote immune cell polarization towards an anti‐inflammatory, non‐cytotoxic phenotype. Reoxygenation injury could revert this phenotype. H, hypoxia, H/R, hypoxia/reoxygenation, A, anoxia, +/‐, increased/decreased by hypo‐EVs compared to normo‐EVs, grey, not studied, MSC, mesenchymal stem cell.

Recently, much effort has been devoted to study more complex signalling pathways of hypo‐EVs between immune cells and other cell types. Guo et al. (2018) demonstrated induced expansion and polarization of myeloid‐derived suppressor cells when they were exposed to hypo‐EVs isolated from glioblastoma cells. These hypo‐EV exposed suppressor cells in turn reduced T cell proliferation, leading to reduced cytotoxicity. Another study from Li et al. (2019) supports this observation that the final anti‐inflammatory effect of hypo‐EVs evident on the T cells could be indirectly mediated by suppressor cells via non‐EV dependent mechanisms. In addition, cancer cells have been shown to promote their own growth and metastasis via hypo‐EV polarized macrophages (Wang et al., 2018). However, reports investigating EVs gathered from cell types other than MSCs and cancer cell‐derived hypo‐EVs are still lacking.

## 7 MicroRNAs IN HYPOXIC EVs

MiRNAs are 21 to 25 nucleotides long, small non‐coding RNAs that regulate gene expression (Bartel, 2004). The classical mechanism for the miRNA mediated gene regulation is the binding of the miRNA to the target mRNA via complementary base pairing. This binding prevents the translation of mRNA into protein, thus inhibiting the expression of the miRNA target gene. MiRNA mediated regulation has been shown to affect most, if not all, biological processes such as the regulation of organ development, cell death, differentiation and proliferation.

MiRNAs are secreted from the cells into the EVs and this secretion is specifically regulated and altered by different conditions such as the activation of T lymphocytes (Villarroya‐Beltri et al., 2013). MiRNAs that were preferentially secreted by T lymphocyte EVs, rather than retained in the cells, were shown to over‐represent RNA motifs that could bind heterogeneous nuclear ribonucleoproteins (hnRNPs). It was suggested that especially sumoylated hnRNPA2B1 bound to the miRNAs and promoted their EV loading, independently of the activation status of the cells. Another protein, synaptotagmin‐binding cytoplasmic RNA‐interacting protein (SYNCRIP), was found to have a similar EV miRNA targeting function in hepatocytes (Santangelo et al., 2016). The specificity of EV miRNA loading is far from clear and would benefit from much more in‐depth mechanistic studies in a variety of different systems to reveal whether there is any specificity of loading of certain miRNAs in different conditions, such as in hypoxia, or are the observed altered miRNA levels in the hypo‐EVs only occurring because of the more general hypoxia‐induced changes in the biogenesis of the EVs.

In the process of miRNA biogenesis, mature single stranded miRNA is produced from the hairpin pre‐cursors, leading to the degradation of the passenger strand (Bartel, 2004). However, in cardiac fibroblasts, although the guide miR‐21‐5p is the more extensively expressed arm of the pre‐miR‐21, it is the passenger miR‐21‐3p that was found to be EV enriched and more extensively secreted to the EVs (Bang et al., 2014). This phenomenon needs further studies in the context of other cell types and miRNAs to evaluate if the secretion of the passenger arm into the EVs could indeed serve as one mechanism for the cell to prefer to express only one of the arms.

As discussed earlier, studies may be biased by the presence of non‐EV contaminants in the EV samples, calling for caution when stating that some miRNAs are carried by the EVs, and that the effect of a certain miRNA is attributable to its transfer from the EVs. In the case of plasma, a major fraction of the RNA in that biological fluid has been shown to be non‐EV associated and there are miRNA specific differences in the extent to which they are packed in the EVs in comparison with the fraction bound to other plasma constituents such as lipoproteins (Karttunen et al., 2019; Vickers, Palmisano, Shoucri, Shamburek, & Remaley, 2011). In addition, while some miRNAs may associate with EVs, the occupancy of less than one copy of a given miRNA per EV has been proposed as likely for even EV enriched miRNAs (Chevillet et al., 2014), encouraging a critical evaluation of how extensive effects these EV associated miRNAs could have in physiological conditions.

### 7.1 Biological pathways affected by hypo‐EV microRNAs

An emerging number of studies have analysed the miRNA contents of hypo‐EVs by qPCR, arrays, or sequencing, revealing an increased release of numerous miRNAs under hypoxic conditions. To take a broader perspective on possible consequences and biological pathways possibly affected by these frequently reported miRNA changes in hypo‐EVs, we carried out an in‐depth analysis of the existing data using bioinformatic tools.

Given the high variation in the study designs reporting changed hypo‐EV miRNA contents, and the lack of freely accessible high throughput data, incorporating all different studies in the analysis proved difficult. To select miRNAs for which there was the highest evidence, and thus strengthening the accuracy of our analysis of the functions of hypo‐EV miRNAs in the recipient cells, we applied specific evidence criteria for the selection of altered hypo‐EV miRNAs and regulated pathways (see Supplementary Methods for the details of analysis and generation of the graphs).

**FIGURE 5:**
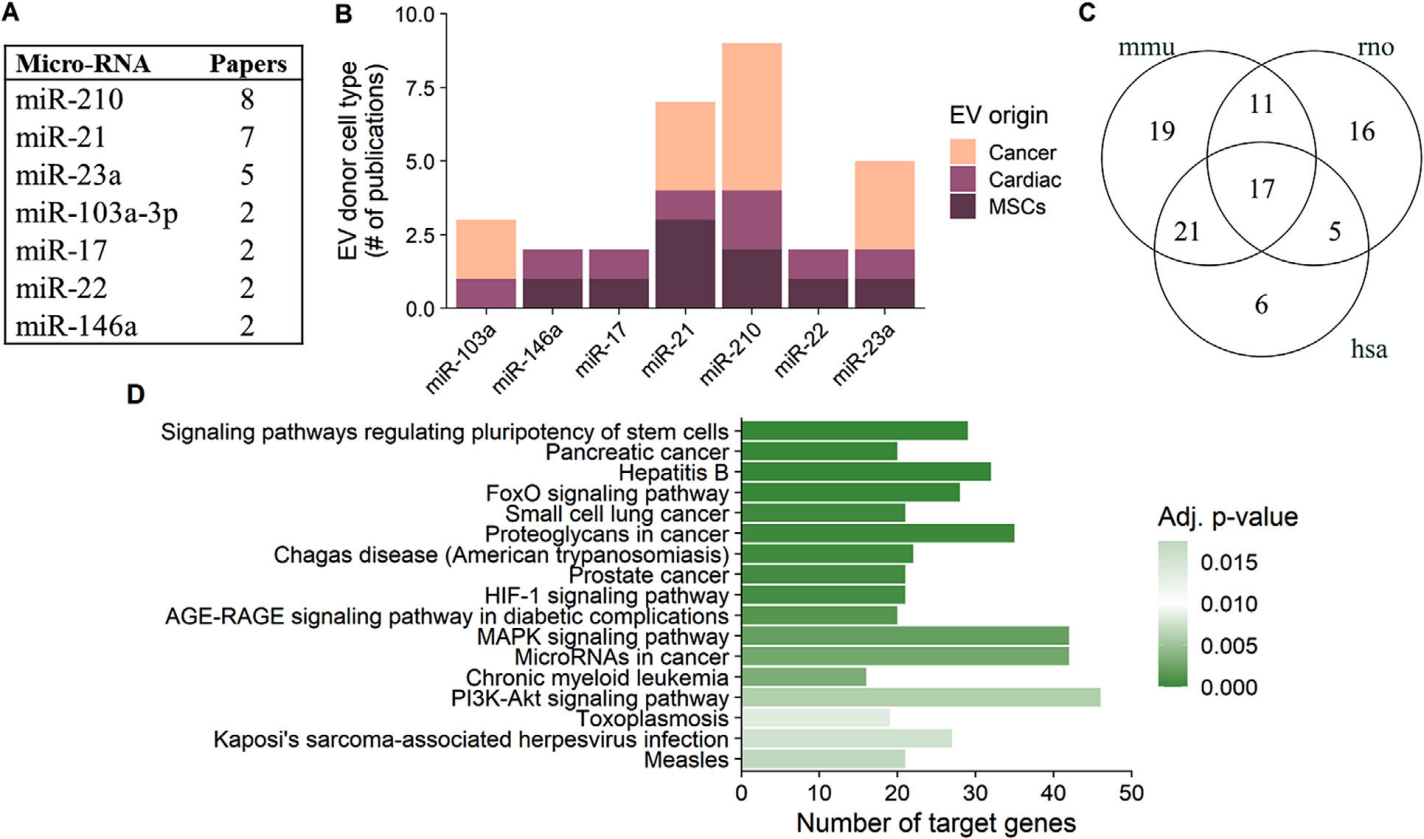
The release of several hypoxia‐related miRNAs is increased in hypoxic extracellular vesicles (EVs). Elevated levels of seven miRNAs have been reported to be released in EVs under hypoxia in more than one publication (a total of 19 publications) (a). In these included studies, EVs were isolated from several donor cell types and the number of publications reporting an increase of specific miRNAs in hypo‐EVs is depicted (b). Separate pathway analysis for each species in the studies, using target genes of altered miRNAs, showed 17 overlapping pathways between human (hsa), mouse (mmu) and rat (rno) (c). These overlapping, significantly enriched, pathways were related to important processes for cell survival in hypoxia (such as FoxO, HIF‐1, MAPK and PI3K‐Akt signalling pathways) and cancer progression (d)

Our analysis revealed seven hypo‐EV enriched miRNAs, listed in Figure 5a, which had been investigated in a total in 19 independent publications. Interestingly, all the miRNAs were increased in hypo‐EVs as compared to normo‐EVs, and furthermore in these experiments, miRNAs had been analysed from EVs isolated from MSCs, cardiac or cancer cells (Figure 5b). As expected, the seven pinpointed miRNAs are generally associated with hypoxic responses at the cellular level (Greco et al., 2014).

Using the validated targets of these hypo‐EV enriched miRNAs, we retrieved 17 biological pathways that were enriched in humans, mice and rats (Figure 5c‐d). The biological processes highlighted in our analysis include cancer related pathways as well as PI3K‐Akt, MAP kinase, HIF‐1 and FoxO signalling pathways, supporting the functional effects of hypo‐EVs in different recipient cell types as these pathways are closely involved in regulating cell proliferation and survival, angiogenesis and migration. However, while hypoxia and induction of HIF‐1 signalling pathways have been previously associated with altered EV release, as far as we are aware, there are no studies which have evaluated the regulatory outcome of a panel of miRNAs carried by hypo‐EVs on HIF‐1 signalling and other hypoxia related pathways. *In vivo*, these hypo‐EV associated miRNAs can originate from several distal hypoxic cell types and contribute to the heterogeneous pool of functional EVs. Thus, the analysis of possible outcomes of several hypo‐EV altered miRNAs together, even if individual miRNAs were to originate from only a certain type of cell, can prove useful when extrapolating the findings to a more physiological, dynamic environment. Our analysis demonstrates for the first time this collection of miRNAs as common regulators of pathways crucial to cellular adaptation to hypoxia, a phenomenon that should be utilized in a wider context in the future.

Consequently, we demonstrate the potential additive effect of increased amounts of several miRNAs in the hypo‐EVs on crucial cellular processes, such as angiogenesis, migration, and immunosuppression, by acting at different levels of the signalling pathways towards common functional outcomes. Furthermore, depending on the cellular context, these outcomes may be detrimental (cancers) or reparative (MI and stroke).

Next, we briefly focus on the literature of each of these pinpointed miRNA to evaluate the relevance of the predicted enriched pathways.

### 7.2 miR‐210

The expression of miR‐210 is directly induced by HIF‐1α that binds to an HRE located in the miR‐210 promoter (Huang et al., 2009). Several studies have described an increase in miR‐210 levels in hypo‐EVs from different cancer cells (Berchem et al., 2016; Jung, Youn, Lee, Kang, & Chung, 2017; King, Michael, & Gleadle, 2012; Umezu et al., 2014; Walbrecq et al., 2020), cardiac cells (Gray et al., 2015; Namazi et al., 2018) and MSCs (Feng et al., 2014; Zhang et al., 2019; Zhu et al., 2018), suggesting hypo‐EV signalling through this miRNA across distinct cell types.

Down‐regulation of anti‐angiogenic targets of miR‐210 drives the expression of VEGF and VEGFR2, leading to angiogenesis (Liu et al., 2012). Indeed, hypoxia‐induced loading of miR‐210 in EVs and delivery to endothelial cells has been shown to be pro‐angiogenic in the context of cancer (Jung et al., 2017; Tadokoro, Umezu, Ohyashiki, Hirano, & Ohyashiki, 2013) and cardiac diseases (Gray et al., 2015; Namazi et al., 2018; Zhu et al., 2018). In cancer, the exposure to hypo‐EV miR‐210 promoted angiogenesis via the suppression of Ephrin‐A3 and protein tyrosine phosphatase non‐receptor type 1 (PTP1B) (Jung et al., 2017; Tadokoro et al., 2013) proteins involved in the regulation of VEGF signalling  (Lanahan et al., 2014; Wang, Liu, Liu, Liu, & Wu, 2015).

VEGFA production is also regulated by the JAK/STAT signalling pathway (Xue et al., 2017). Interestingly, the signalling in this pathway is enhanced by miR‐210 through another direct target, suppressor of cytokine signalling 1 (SOCS1), a negative regulator of the JAK/STAT pathway (Yoshimura, Naka, & Kubo, 2007; Zhang et al., 2019). These observations support the hypothesis that miR‐210 acts as a pro‐angiogenic factor through several distinct pathways, promoting reparative functions after MI (Namazi et al., 2018; Zhu et al., 2018) and enhancing the invasiveness and growth of cancerous tissue (Zhang et al., 2019).

### 7.3 miR‐21

Like miR‐210, the expression of miR‐21 is partly regulated by HIF‐1α and HIF‐2α (Li et al., 2016). A hypoxic microenvironment stimulates miR‐21 expression in tumour cells and its release in EVs (Guo et al., 2018; Li et al., 2016). Indeed, serum EV miR‐21 levels showed a positive correlation with the hypoxia status of tumours and lymph node metastasis (Li et al., 2016), suggesting that hypoxia is an important regulator of miR‐21.

Induction of miR‐21 expression at the cellular level has been demonstrated to promote proliferation and to suppress apoptosis (Zhang et al., 2017). Similar functions of hypo‐EV miR‐21‐5p from bone marrow‐derived MSCs have been described, evidence for a tumour promoting effect (Ren et al., 2019). Down‐regulation of PTEN expression, via direct targeting by hypo‐EV delivered miR‐21, has been shown to promote the immunosuppressive functions of tumour associated macrophages (Ren et al., 2019) and myeloid‐derived suppressor cells (Guo et al., 2018; Li et al., 2019), likely leading to cancer progression. In addition, miR‐21 was claimed to induce cell migration and invasion, possibly promoting cancer metastasis (Li et al., 2016).

Upregulation of miR‐21 has been linked to cardioprotective effects in cardiovascular diseases (Zhang et al., 2017). A role for miR‐21 in pulmonary hypertension has been suggested, as hypoxia induced the expression of miR‐21 in pulmonary artery smooth muscle cells (Sarkar et al., 2010). Indeed, pulmonary hypoxia may induce miR‐21 release into circulating serum EVs, as shown in idiopathic pulmonary fibrosis (Makiguchi et al., 2016), a condition linked to hypoxia (Plantier et al., 2018). Together these studies indicate that the increase of miR‐21 in hypo‐EVs goes beyond cancer, highlighting the importance of studying this topic outside the realms of cardiovascular and cancer disorders.

### 7.4 miR‐23a

The third most widely hypo‐EV associated miRNA in the literature, miR‐23a, differs from miR‐210 and miR‐21 by indirectly regulating cellular levels of HIF‐1α (Hsu et al., 2017), while the two others are mainly regulated downstream of this transcription factor. miR‐23a reduces the expression of PHD1/2 (Hsu et al., 2017), proteins normally hydroxylating HIF‐1α instead directing it to proteasomal degradation (Pientka et al., 2012). A reduced expression of PHD1/2 leads to an increase of HIF‐1α signalling, higher VEGF expression and augmented angiogenesis in hypo‐EV miR‐23a exposed cancer cells (Hsu et al., 2017). Another miR‐23a target, SIRT1, may contribute to the angiogenesis promoting effect of this miRNA (Sruthi et al., 2018). However, contradicting reports exist on the role of SIRT1 in inducing or inhibiting angiogenesis (Gao et al., 2016; Kunhiraman et al., 2017; Potente et al., 2007; Zhang et al., 2013), these discrepancies may be due to parallel signalling pathways that become activated along with SIRT1.

The suppression of NK cell‐mediated anti‐tumour immune responses by hypo‐EVs may be partly elicited through miR‐23a inhibited CD107a expression (Berchem et al., 2016), a marker of NK activity (Alter, Malenfant, & Altfeld, 2004). Moreover, miR‐23a enhances cancer cell transendothelial migration by targeting ZO‐1 (Hsu et al., 2017), a junctional adaptor protein which plays a central regulatory role in the control of endothelial barrier formation (Tornavaca et al., 2015).

Interestingly, a recent paper demonstrated that miR‐23a‐5p was reduced in hypo‐EVs from melanoma cells (Walbrecq et al., 2020). The miRNA23a‐5p (previously known as miRNA‐23a*) is the strand that usually is degraded during the biogenesis of the miRNA (Hammond, 2015). However, the miRNA/miRNA* ratio can vary during carcinogenesis (Tsai et al., 2016; Zhang et al., 2019), underscoring the relevance of considering both miRNA strands in the analysis of the miRNAs carried by EVs.

Studies of hypo‐EV miR‐23a outside oncogenic disorders remain few in number. The cardioprotective role of miR‐23a‐3p in hypo‐EVs has been demonstrated by Zhang et al. (2017). The levels of miR‐23a were found to be decreased in the serum of patients suffering from type 2 diabetes (Yang et al., 2014) and to be related to treatment response (Catanzaro et al., 2018). In diabetes, hypoxia has been implicated in beta‐cell damage (Sato et al., 2011), suggesting that miR‐23a has relevance also in non‐oncogenic pathologies. Interestingly, our pathway analysis pinpointed the AGE‐RAGE signalling pathway in diabetic complications as an enriched pathway, suggesting that in the context of diabetes, hypo‐EVs could be an intriguing focus for future studies.

### 7.5 Other miRNAs

There is a paucity of publications describing other hypo‐EV associated miRNAs. Hypo‐EVs from tumour cells have been described to carry high levels of miR‐103a (Hsu et al., 2017; Hsu et al., 2018). miR‐103a can be transferred to monocytes where it can inhibit the expression of PTEN by direct targeting, leading to the activation of PI3K/Akt and STAT3 signalling pathways, finally resulting in a switch towards the tumour‐promoting macrophage polarization (Hsu et al., 2018). The hypo‐EV‐contained miR‐22‐3p, miR‐146a‐5p and miR‐17 may exert cardioprotective roles (Gray et al., 2015; Zhang et al., 2017). Hypo‐EVs from bone marrow‐derived MSCs can be transferred to cardiomyocytes and act as cardioprotective mediators via the downregulation of Mecp2 by miR‐22, reducing apoptosis *in vitro* and improving post‐MI fibrosis *in vivo* (Feng et al., 2014).

Interestingly, the study of Cui et al. (2018) analysed the role of hypo‐EVs in the context of cognitive decline and detected the presence of some of these previously mentioned microRNA. Hypo‐EVs from bone marrow‐derived MSCs were found to contain increased levels of several miRNAs, including miR‐210, miR‐21, miR‐23a, miR‐17 and miR‐146a. The infusion of these hypo‐EVs ameliorated spatial learning and memory impairments in a mouse model of Alzheimer's disease, by alleviating Aβ accumulation, increasing synaptic protein expression, inhibiting the activation of astrocytes and microglia, as well as by decreasing the levels of pro‐inflammatory compounds while increasing those with anti‐inflammatory effects.

## 8 OTHER RNAs AND PROTEINS IN HYPOXIC EVs

Data on other molecules than miRNAs in hypo‐EVs are more limited. However, an increase in the enzymatic activity in hypo‐EVs compared to normo‐EVs has been reported (Jong et al., 2016, Mleczko et al., 2018). In particular, the matrix metalloproteinase activity has been shown to be increased in hypo‐EVs collected from first trimester cytotrophoblasts (Salomon et al., 2013), nasopharyngeal carcinoma (Shan et al., 2018) and prostate cancer cells (Ramteke et al., 2015). In addition, the release of cytokines (Ramteke et al., 2015; Salomon et al., 2013; Yu et al., 2012), VEGF (Salomon et al., 2013) and HIF1α (Burnley‐Hall et al., 2017) proteins has been reported. Indira Chandran et al. (2019)) utilized proteomics and analysed hypo‐ and normo‐EVs from glioblastoma cells and confirmed the increased release of IGFBP3 and CA9 in hypoxia. They showed enrichment of HIF signalling pathway proteins in hypo‐EVs and also validated this altered release at the transcriptional level. A lack of stable proteins for normalization was emphasized as an important limitation. Unfortunately, this limitation currently applies to all EV studies and in the context of hypoxia, may be especially important as hypoxia may increase the release of EVs.

## 9 DISCUSSION

Regardless of the major differences in study designs and technical aspects, the literature on the effects of hypoxia on EV release appears to be highly consistent regarding the functions of hypoxic EVs in the recipient cells. These functions involve increased invasiveness and migration, proliferation, angiogenesis and immunomodulation. Some opposing effects have also been reported which may be due to different cell types, type of stimuli and other experimental parameters. Due to the relatively small number of closely identical studies, definite conclusions cannot be made, but the current information serves as a promising platform from which to design future studies to replicate the findings with more state‐of‐art methods to achieve a better characterization of the EVs. Importantly, we would encourage studies also outside the MSC, cancer or cardiovascular fields as current knowledge in these contexts is very limited.

A growing number of studies have highlighted the potential contribution of EVs to human disorders involving pathogenic hypoxia. The analysis of the hypo‐EV cargo is essential if one wishes to elucidate the functions of these EVs in the context of complex disorders and to better understand the multifaceted communication among different cell types in response to hypoxia. MiRNAs can directly affect the behaviour of cells by targeting specific transcripts involved in a variety of cellular processes. Interestingly, our bioinformatic analysis of several hypo‐EV associated miRNAs across species revealed how they may regulate many important pathways involved in cellular adaptation to hypoxia, strongly supported by the existing studies on individual miRNAs.

Since hypo‐EVs can affect target cells in different ways as compared to normo‐EVs, their inclusion in the studies of novel therapeutic options should be taken into account, for example, in the context of cardiac disorders, where there is convincing evidence pointing to their cardioprotective function. On the other hand, modifications of EV membrane could be exploited to target drug loaded EVs to specific target tissues or cells which could reduce the risk of side effects or increase the potency of drugs. In addition, the characteristics of EVs suggest that they may be a tempting target for biomarker development as EVs originating from a certain cell type could be identified and quantified, i.e. providing biomarkers with improved disease specificity.

Future studies will benefit greatly from the expected technological developments in reference materials, measurement of single EVs and improved purity of EVs, as well as in the detection of different EV subpopulations. By taking advantage of these advances, hypo‐EV studies should focus on defining in more detail the effect of cell death on the release of EVs and on the co‐purifying of non‐EV contaminants under hypoxic conditions. Furthermore, the clarification of the many miRNAs in the EVs’ cargos, as well as the effects of the EV‐derived miRNAs in the recipient cells need further evaluation. The use of databases such as EV‐TRACK (Van Deun et al., 2017) should lead to rapid progress in the EV field by promoting the careful and open reporting of all experimental parameters as recommended in the updated MISEV2018 guidelines.

## CONFLICT OF INTEREST

The authors report no conflict of interest.

## GEOLOCATION INFORMATION

Kuopio, Finland.

## Supporting information

Supplementary information
