# Supplementary material for: Hypoxia and extracellular vesicles: A review on methods, vesicular cargo and functions
Source: J Extracell Vesicles. 2020 Nov 14;10(1):e12002. doi: 10.1002/jev2.12002 (PMC7710128; doi:10.1002/jev2.12002)
Supplement: Supplementary file 1 — Supplementary information [file JEV2-10-e12002-s001.docx]

# Supplementary methods

## Retrieving microRNAs altered in hypoxic EVs

The relevant publications were searched from PubMed using “extracellular vesicle”, “exosome” or “microvesicle” and “hypoxia” or “ischemia” search words and screening for suitable articles based on the title. Studies published before February 28th, 2020 were included. Publications comparing the level of miRNAs between EVs from hypoxic and normoxic cells were selected. To include miRNAs with highest evidence to the pathway analysis, miRNAs that were shown to be altered in the same direction in at least two independent studies were selected. MiRNAs detected by high throughput methods were additionally validated by another method if they were to be included in the analysis.

## Pathway analysis

Validated miRNA targets were retrieved from miRTarBase [1] and enriched pathways were obtained using the KEGG Pathway database. Since targets of the same miRNA may differ between species due to non-conserved 3’ UTR sequences, we performed the pathway analysis separately for human, mouse and rat validated miRNA targets, the three species examined in the included studies. To increase the applicability across species, filtering for pathways found to be enriched in all studied species was applied. For data visualization, R packages VennDiagram and ggplot2 were used.

## References

[1] Chou C-H, Shrestha S, Yang C-D, et al. miRTarBase update 2018: a resource for experimentally validated microRNA-target interactions. Nucleic Acids Res. [Internet]. 2018;46:D296–D302. Available from: http://academic.oup.com/nar/article/46/D1/D296/4595852.
